# Bilateral knee pigmented villonodular synovitis in a young adult: Radiologic diagnosis and surgical approach

**DOI:** 10.1016/j.radcr.2024.09.095

**Published:** 2024-10-17

**Authors:** Adnane Lachkar, Abdeljaouad Najib, Hicham Yacoubi

**Affiliations:** a Faculty of Medicine and Pharmacy, Mohammed First University, Oujda, Morocco; b Department of Traumatology, Orthopedic Mohammed VI University Hospital, Oujda, Morocco

**Keywords:** Pigmented villonodular synovitis, Bilateral knee involvement, Synovectomy, Synovial proliferative disorder

## Abstract

Pigmented villonodular synovitis is a rare proliferative disorder of the synovial joints, characterized by synovial thickening and hemosiderin deposition. It predominantly affects the knee joint, with bilateral involvement being exceedingly rare. We present a case of diffuse bilateral pigmented villonodular synovitis in a 20-year-old female, initially presenting with left knee pain and swelling, diagnosed via MRI. Following an open total synovectomy, the patient developed postoperative stiffness, requiring arthroscopic arthrolysis. Subsequently, similar symptoms emerged in the right knee, leading to a second synovectomy and subsequent arthrolysis. Despite the challenges associated with managing this condition, including a high recurrence rate and postoperative stiffness, the patient showed significant improvement 1 year postoperatively. This case underscores the importance of thorough synovectomy and highlights the potential need for further surgical intervention to address complications such as stiffness.

## Introduction

Pigmented Villonodular Synovitis (PVNS) is a rare disorder affecting the synovial joints, characterized by synovial thickening and hemosiderin-laden masses, which confer its distinctive pigmentation [1]. The incidence rate is approximately 1.8 cases per million people annually [2]. While PVNS is typically benign, malignant transformation is a rare possibility [3]. This condition can affect various joints across all age groups, but it predominantly occurs in the knees of young adults [2]. Bilateral involvement, whether simultaneous or sequential, is exceptionally rare, with only a few documented cases in the literature [4,5]. This case report describes an unusual instance of diffuse bilateral PVNS affecting both knees in a single young patient.

## Case presentation

**Fig. 1:**
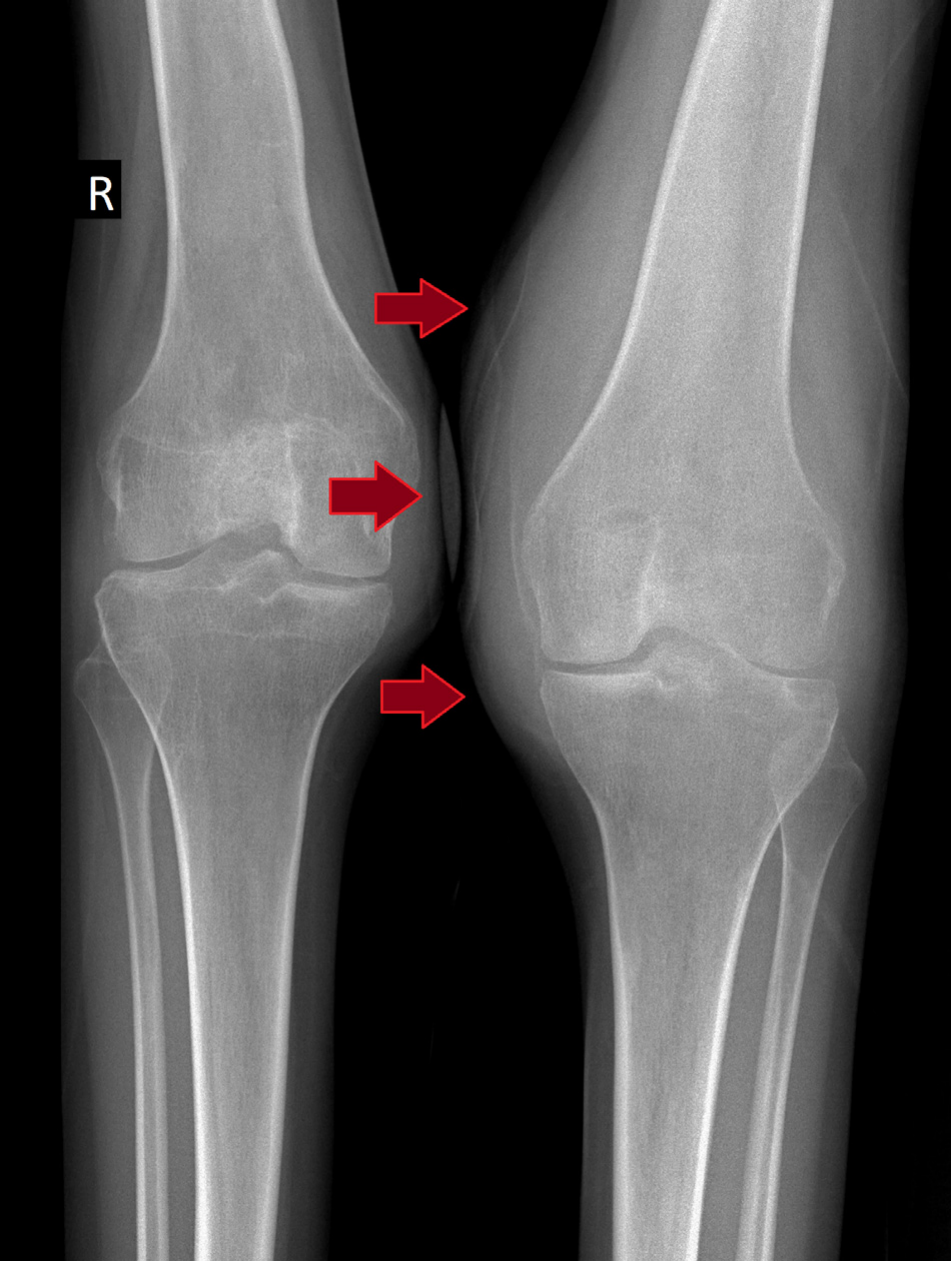
Anteroposterior X-ray showing a soft-tissue mass in the left knee. The arrows indicate the increased soft-tissue shadow in the left knee.

**Fig. 2:**
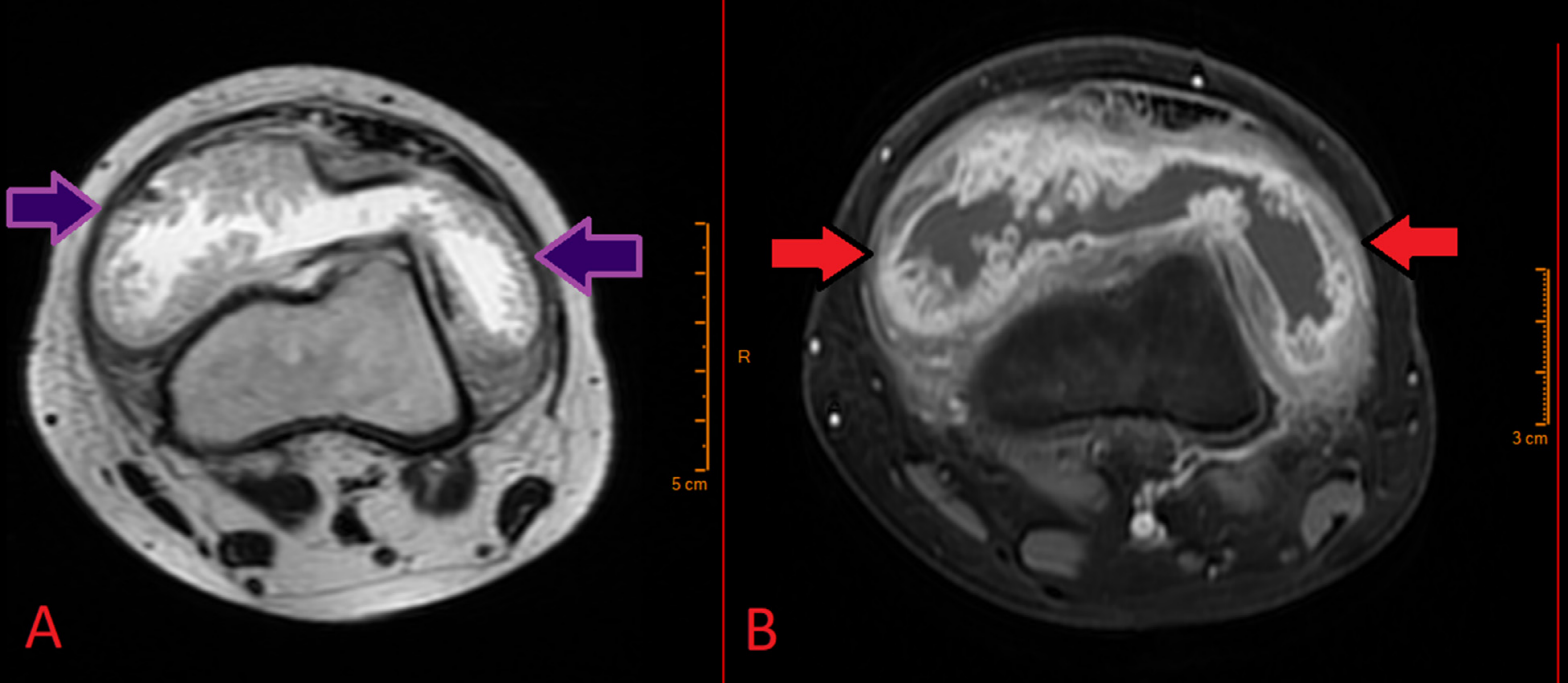
Axial MRI of the right knee demonstrating diffuse intra-articular pigmented villonodular synovitis (PVNS). (A) T2-weighted image showing a multinodular lesion with low signal intensity due to the presence of hemosiderin deposits. The lesion is characterized by diffuse synovial thickening and irregular nodular areas. (B) T1-weighted, fat-saturated postcontrast sequence revealing enhanced synovial proliferation with marked contrast uptake indicating active disease. The arrows highlight the extent of synovial thickening and nodularity, with hemosiderin deposits visible as areas of low signal intensity.

A 20-year-old female presented with intermittent pain in the left knee, exacerbated by physical activity, with no history of trauma. Physical examination revealed significant swelling of the left knee, a restricted range of motion with approximately 100 degrees of flexion, a 5-degree flexion contracture, and mild peripatellar tenderness. Initial radiographs showed a soft-tissue mass in the left knee (Fig. 1). An MRI, conducted due to persistent pain, revealed a multinodular synovial lesion with typical features of diffuse pigmented villonodular synovitis (PVNS). The lesion appeared hypo-intense on T2-weighted images, reflecting hemosiderin deposition, and iso- to hypo-intense on T1-weighted images. The MRI also showed synovial thickening and postcontrast enhancement, consistent with active synovial proliferation. These findings, combined with hemosiderin deposits and mild joint effusion, confirmed the diagnosis of diffuse PVNS (Fig. 2).

**Fig. 3:**
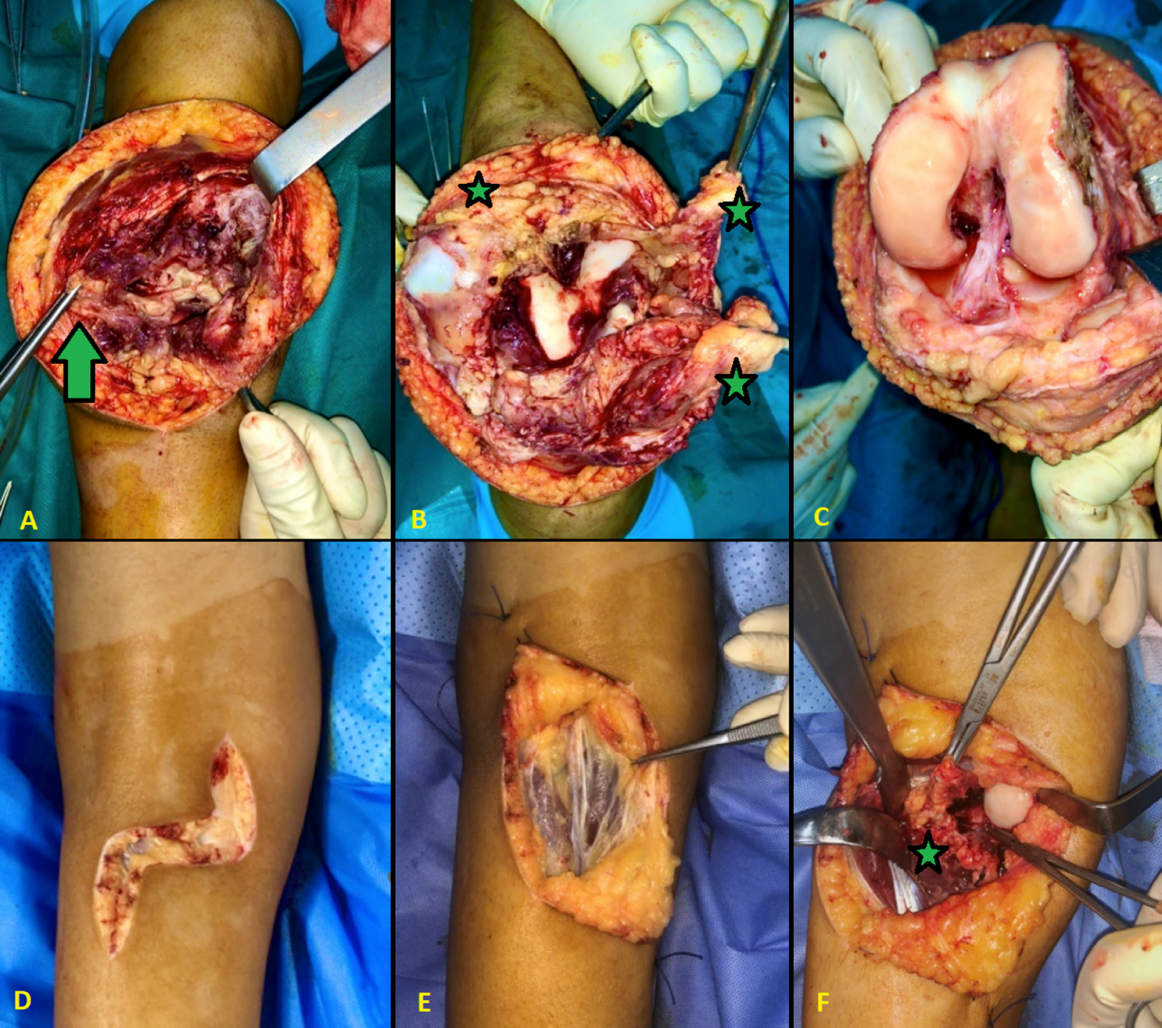
Perioperative images of the open approach synovectomy. (A) Anterior approach; superficial dissection. The arrow indicates the macroscopic appearance of the synovitis. (B) Anterior approach; exposure of the PVNS. The stars indicate the villonodular synovitis, which appears as a chamois yellow. (C) Anterior approach; results after anterior synovectomy. (D) Posterior approach; superficial dissection. (E) Posterior approach; exposure of the popliteal neurovascular bundle (indicated by the arrow). (F) Posterior approach; posterior synovectomy. The star indicates the exposed synovitis prepared for resection.

The treatment plan involved an open total synovectomy of the left knee to completely excise the PVNS lesions. This procedure included a large anterior approach for intra-articular synovectomy, followed by a posterior approach (Fig. 3). Due to the patient's young age, adjuvant radiosynoviorthesis was not recommended. Histological examination of the excised tissue confirmed the diagnosis of PVNS. Despite undergoing a 6-month rehabilitation program, the patient experienced persistent stiffness and a limited range of motion (80 degrees of flexion in the left knee).

**Fig. 4:**
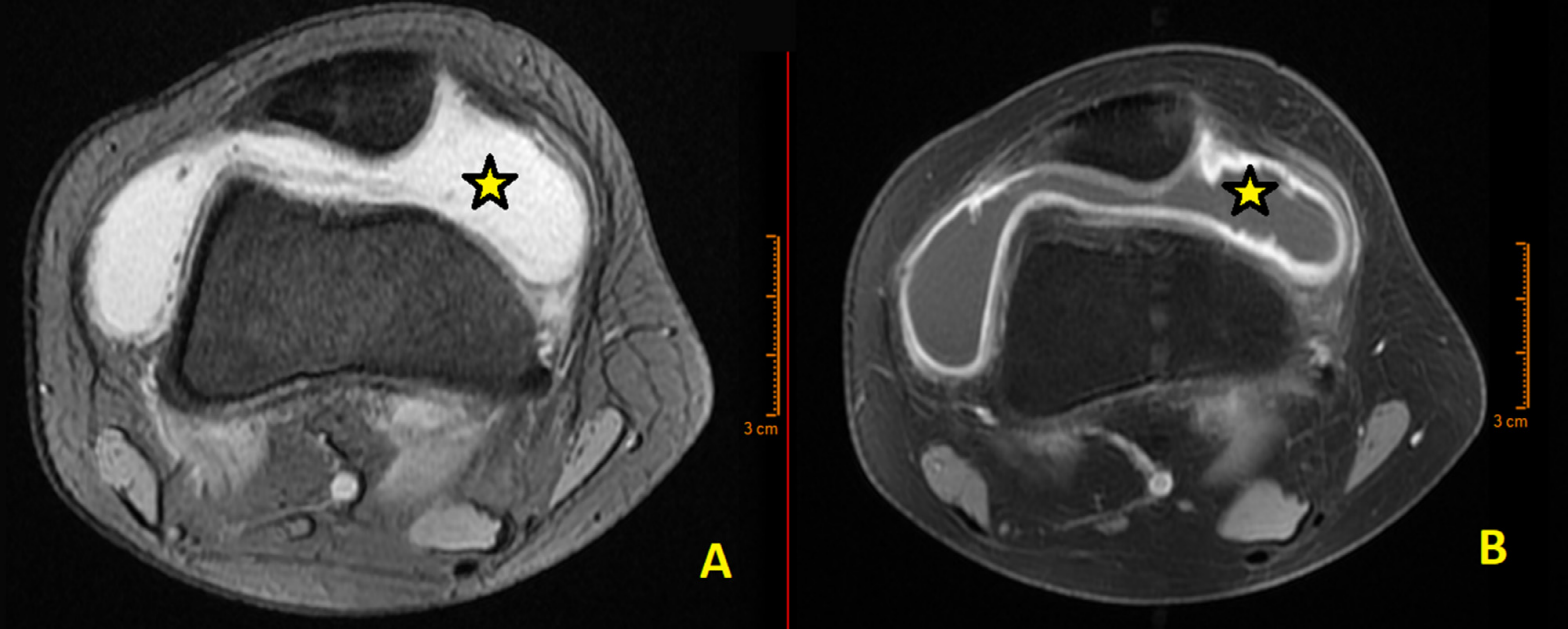
Axial MRI of the left knee demonstrating diffuse intra-articular Pigmented Villonodular Synovitis (PVNS). (A) T2-weighted multi-echo gradient image showing a multinodular lesion with low signal intensity due to hemosiderin deposits. The image reveals extensive synovial thickening and nodular formations, with the hemosiderin deposits contributing to the observed signal drop-out effect typical of PVNS. (B) T1-weighted, fat-saturated postcontrast sequence displaying enhanced synovial proliferation with marked contrast uptake, highlighting the extent of synovial thickening and nodularity. The stars indicate the villous and nodular lesions, which are prominent due to the contrast enhancement and low signal intensity areas from hemosiderin deposits.

**Fig. 5:**
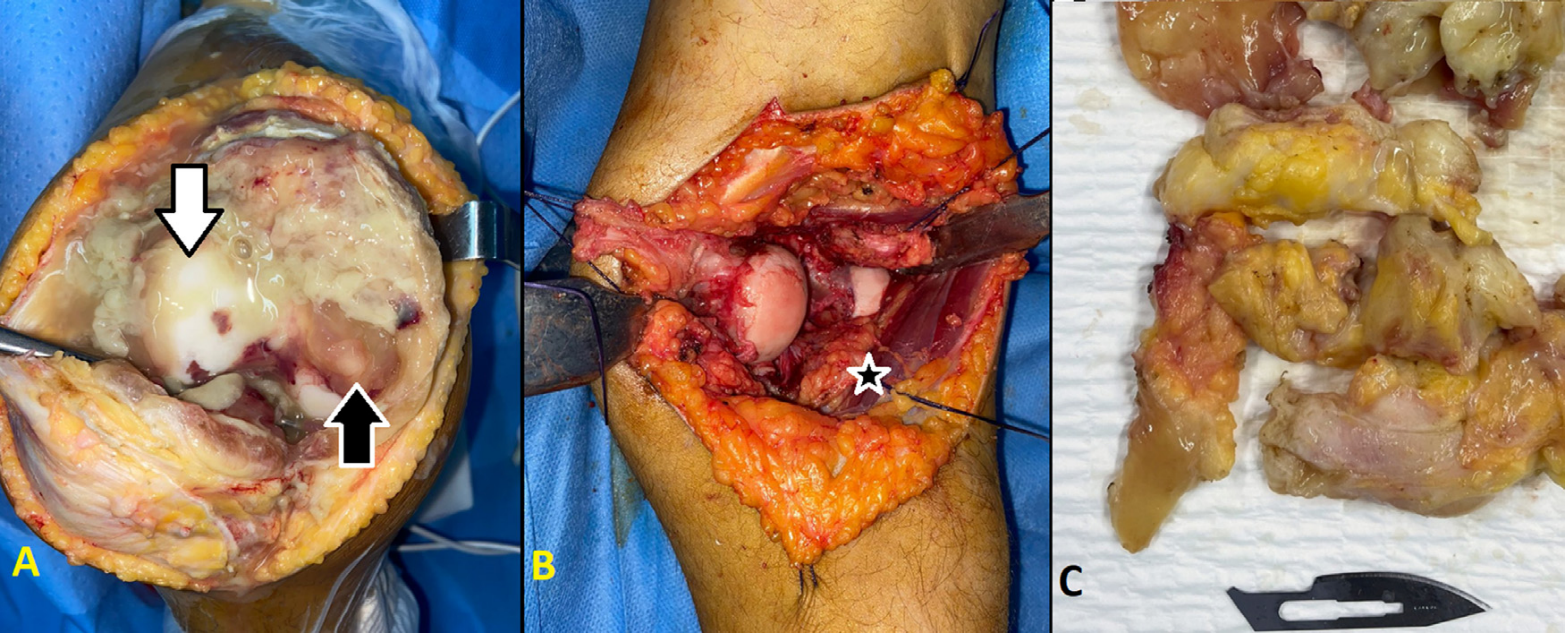
Perioperative images of the open approach synovectomy of the right knee. (A) Anterior approach; the black arrow indicates the pigmented villonodular synovitis; the white arrow indicates the cartilage of the lateral femoral condyle. (B) Posterior approach; the star indicates the intra-articular Pigmented Villonodular Synovitis. (C) Excised synovial tissue.

During this period, the patient developed pain and swelling in the right knee. Radiographs and MRI revealed lesions consistent with diffuse PVNS (Fig. 4). Surgical intervention was performed to completely remove these lesions (Fig. 5), and histological analysis confirmed the diagnosis. Three months later, an arthroscopic arthrolysis was performed on the left knee to address stiffness, followed by a similar procedure on the right knee 3 months later due to postsynovectomy stiffness. At the 1-year follow-up, the patient's symptoms had significantly improved, with both knees regaining flexion. One year after the final surgery, the patient reported satisfactory resolution of symptoms.

## Discussion

The first documented case of PVNS was reported by Chassaignac in 1852 [6]. In 1941, Jaffe [7] provided a comprehensive description of the disease, highlighting key features such as lipid-laden macrophages, multinucleated giant cells, and hemosiderin deposits within a fibrous stroma. Histologically, PVNS is characterized by lipid-laden macrophages, multinucleated giant cells, hemosiderin deposits, polyhedral histiocytic or spindle cells, and proliferating round cells [2]. The diffuse form of PVNS is notably associated with a high recurrence risk, leading some to consider it a neoplastic process [8]. This is supported by the presence of chromosomal abnormalities, such as trisomy 5 and 7, clonal DNA rearrangements, and rare instances of malignant transformation [9].

Hirohata proposed that localized disruptions in lipid metabolism could contribute to the development of PVNS [10]. Although trauma has been considered a potential etiological factor, subsequent studies have not substantiated this hypothesis. Notably, patients with hemophilic arthropathy exhibit histological features resembling PVNS, including lobular synovitis and hemosiderin deposits [2,9].

The clinical presentation of diffuse PVNS is typically insidious, with symptoms progressively worsening over months to years before medical consultation [2]. This slow progression complicates the determination of the disease's timeline, particularly in cases involving multiple joints. In bilateral PVNS, the interval between the onset of involvement in the first and subsequent joints can vary significantly, from a few months to 3 years, as observed in our case with a 12-month interval [4,5]. Multifocal PVNS may present with even longer intervals, up to 17 years, suggesting a potential genetic predisposition [5]. However, the possibility of metastasis or new disease formation cannot be entirely excluded [3].

Initial radiographs may appear normal or show signs of joint effusion, soft-tissue mass, and bony erosion. These well-defined extrinsic erosions, often with rim sclerosis, may be observed on both sides of the joint [2]. They likely result from contact or mass effect on adjacent bone and are more common in smaller, lower-capacity joints [11]. To differentiate PVNS from conditions such as post-traumatic or degenerative intra-articular bodies or synovial osteochondromatosis, it is crucial to note that intra-articular lesions in PVNS typically lack calcification. Computed tomography (CT) scans may reveal features similar to radiographs, including synovial thickening and increased attenuation due to hemosiderin deposition [11]. Magnetic resonance imaging (MRI) offers more detailed visualization of erosions, synovial thickening, joint effusion, and soft-tissue masses compared to radiographs or CT scans [12]. In diffuse PVNS, MRI typically shows multinodular synovial thickening with low or intermediate signal intensity across all pulse sequences. A distinctive feature of PVNS is the “blooming” effect observed on gradient echo sequences, resulting from the magnetic susceptibility artifact caused by hemosiderin [11,12].

Complete synovectomy, whether performed via open or arthroscopic methods, is crucial for the management of diffuse PVNS due to the high recurrence risk [9]. Some authors advocate for the open approach to ensure more thorough removal of the synovial tissue. De Ponti et al. [13] observed a 20% recurrence rate following total synovectomy and a 50% recurrence rate after partial synovectomy in their study involving 15 patients over an average follow-up period of 5 years. These results underscore the importance of comprehensive synovectomy to reduce recurrence rates in diffuse PVNS. In cases with extra-articular involvement, arthroscopic techniques alone may be insufficient. Sharma et al. [14] reported a 25% recurrence rate using a combination of arthroscopic and dorsal open approaches in select cases. Consequently, some practitioners prefer fully open procedures to address concomitant extra-articular manifestations effectively. However, extensive open approaches can lead to postoperative stiffness, potentially necessitating surgical arthrolysis, as observed in our case. This procedure can be challenging due to fibrosis and scar tissue formation, and may lead to less favorable outcomes with ongoing pain and stiffness [9].

Recent studies indicate that adjuvant radiosynoviorthesis may benefit the management of diffuse and recurrent PVNS [15], [16], [17]. Nevertheless, the use of intra-articular radioisotope injection as an adjunctive treatment remains debated due to potential risks, including infection, malignancy induction, and radionecrosis of surrounding soft tissues [15,17].

## Conclusions

In summary, PVNS is a rare but clinically significant proliferative disorder of the synovial tissue, primarily affecting large joints such as the knee. Histological examination reveals characteristic features, including lipid-laden macrophages and hemosiderin deposits, which highlight its potential for neoplastic behavior. The condition often presents insidiously, complicating early diagnosis, particularly in cases involving diffuse or multifocal forms. MRI is essential for detailed imaging and accurate disease assessment. Treatment through synovectomy, whether open or arthroscopic, poses challenges due to high recurrence rates, especially in diffuse PVNS. Although adjuvant radiosynoviorthesis shows potential benefits, its use remains debated due to associated risks. Effective management requires thorough synovectomy and vigilant postoperative care to minimize recurrence and improve patient outcomes.

## Patient consent

The patient's written informed consent for the publication of this case report and accompanying images has been obtained and can be provided upon request by the Editor-in-Chief.
